# Assessing thrombogenesis and treatment response in congenital thrombotic thrombocytopenic purpura

**DOI:** 10.1002/jha2.178

**Published:** 2021-02-28

**Authors:** Ferras Alwan, Chiara Vendramin, Ulrich Budde, Ri Liesner, Alice Taylor, Mari Thomas, Bernhard Lämmle, Marie Scully

**Affiliations:** ^1^ Department of Haematology University College London Hospital London UK; ^2^ Haemostasis Research Unit University College London London UK; ^3^ Medilys Laboratory Coagulation Asklepios Hospital Altona Hamburg Germany; ^4^ Haemophilia Comprehensive Care Centre Great Ormond Street Hospital for Children NHS Trust London UK; ^5^ NIHR Great Ormond Street Hospital Biomedical Research Centre London UK; ^6^ Department of Haematology University College London Hospital Cardiometabolic Programme‐NIHR UCLH/UCL BRC London UK; ^7^ Department of Hematology and Central Hematology Laboratory Inselspital Bern University Hospital University of Bern Bern Switzerland; ^8^ Center for Thrombosis and Hemostasis University Medical Center Mainz Mainz Germany

**Keywords:** congenital TTP, prophylaxis, shear flow, upshaw schulman syndrome

## Abstract

Despite clinical remission and normal platelet counts, congenital TTP (cTTP) is associated with non‐overt symptoms. Prophylactic ADAMTS13 replacement therapy such as plasma infusion (PI) prevents acute episodes and improves symptomatology. There is no current method to investigate disease severity or monitor the impact of treatment. We utilize a dynamic high shear flow assay to further understand disease pathophysiology and determine the impact of cTTP on symptomatology and therapy, despite normal platelet counts. Whole blood, under high shear, was run over collagen‐coated channels, causing platelet adhesion to von Willebrand factor (VWF) multimers. The resulting surface coverage by platelet‐VWF thrombus was assessed. The normal range was 6–39% in 50 controls. Twenty‐two cTTP patients with normal platelet counts were evaluated. Median pre‐treatment surface coverage was 89%, and PI reduced coverage to a median of 44% (*p* = 0.0005). Patients taking antiplatelets had further reduced coverage when combined with PI and improved non‐overt symptoms such as headache, lethargy, and abdominal pain in 100% of patients compared to 74% with PI alone (*p* = 0.046). We use a dynamic assay to report increased in vitro platelet adhesion and aggregation and additionally demonstrate significantly decreased thrombi following PI, with levels in the normal range levels achieved in patients taking additional antiplatelet therapy.

## 1 INTRODUCTION

Congenital TTP (cTTP) is an ultra‐rare disorder defined by an inherited deficiency of the von Willebrand factor (VWF) cleaving enzyme *a disintegrin and metalloproteinase with a thrombospondin type 1 motif, member 13* (ADAMTS13) leading to the presence of highly adhesive ultra large VWF multimers. Acute events present similarly to immune‐mediated TTP with thrombocytopenia and microangiopathic hemolytic anemia, and there is broad consensus that such events should be treated with ADAMTS13 rich blood products such as fresh frozen plasma (FFP) infusion [1] (PI). While acute events are less frequent in patients receiving PI, there is increasing evidence that cTTP should be treated as a chronic disease with stroke, cardiac, and renal sequelae among the long‐term complications reported [2, 3, 4] in untreated patients.

Regular prophylactic treatment decreases long‐term morbidities [2, 5], but there is little agreement on treatment regimens due to a lack of evidence in this ultra‐rare condition and an inability to gauge disease severity. The most obvious tools available to physicians are frequently unhelpful: platelet and hemoglobin counts are usually within the normal laboratory range outside of acute events [4], and there is a poor correlation between ADAMTS13 activity levels, even down to <1% of normal activity [6]. There is considerable heterogeneity in ADAMTS13 mutations causing cTTP, with over 150 mutations described [7] with differing overt disease onset [8, 9] and severity associated with different mutations [10]. To date, the largest studies in this area show no strong correlation between specific genetic mutations and disease severity [4, 6]. Further difficulties, both conceptual and practical, are encountered if patients are commenced on prophylactic treatment. Non‐overt symptoms including lethargy, headaches, and abdominal pain, commonly reported by cTTP patients and recognized as disease related [4], are improved by ADAMTS13 replacement, but their pathophysiology has hitherto been unclear, as they occur despite normal hemoglobin and platelet counts. Rationalizing treatment intensity and frequency is limited by current ADAMTS13 replacement therapies that only partially correct activity levels [11], and in the absence of normalization, there is only limited evidence on appropriate targets [11]. The assays currently used to assess ADAMTS13 activity lack sensitivity to measure very low levels, relevant as the half‐life of ADAMTS13 after FFP infusion is around 3–5 days [11, 12, 13], and infusions at a frequency of more than once weekly are impractical for most patients.

Current ADAMTS13 assays operate under static conditions [14, 15, 16]. However, shear flow is crucial to the entire process as the VWF A2 domain bond cleaved by ADAMTS13 is only exposed by shear stress [17]. Additionally, static assays do not take into account the effect of flow on different ADAMTS13 mutations, which can influence VWF binding when exposed to shear flow conditions [18, 19]; or the effect of VWF levels, a factor previously found to affect thrombus formation in cTTP [20].

In this study we utilize a dynamic shear flow assay to assess VWF and platelet binding to collagen coated channels as a surrogate marker for thrombus formation in cTTP patients with normal blood counts and no evidence of active thrombotic microangiopathy, to better understand the disease pathophysiology and evaluate the impact of current treatment response and therapeutic options.

## 2 METHODS

### 2.1 Patient selection

All patients with cTTP under the care of a single tertiary center during a 1‐year period were included. All patients were consented (Medical Research Ethics Committee Numbers 08/H0810/54 and 08/H0716/72). cTTP was defined as patients with ADAMTS13 protease activity below 10 IU/dL (FRETS VWF‐73 assay, normal range 60–146 IU/dL), no evidence of inhibitory anti‐ADAMTS13 IgG antibodies (normal range < 6%), and the identification of a biallelic ADAMTS13 mutation causing cTTP. Patient samples were additionally analyzed for complete blood count, VWF antigen, and VWF activity.

### 2.2 ADAMTS13 assays

ADAMTS13 activity was measured using the FRETS VWF‐73 method [14], and an ELISA method was used to quantify anti‐ADAMTS13 IgG antibodies [21]. Genetic mutations were identified using Sanger sequencing after polymerase chain reaction amplification with the use of custom oligonucleotide primers.

### 2.3 Shear flow‐based assay

A shear flow‐based assay was developed using a *Cellix VenaFlux* (Cellix Ltd, Dublin, Ireland) semi‐automated microfluidic platform to mimic hemostatic capacity under shear flow. Citrated, whole blood from cTTP patients was used to undertake analysis of VWF adsorption to type I collagen, subsequent platelet adhesion, and platelet aggregation.

cTTP samples associated with normal full blood counts were analyzed within 4 h of venesection. Each sample was run in duplicate, and a mean value was taken. Citrated blood was treated with DiOC_6_ to achieve platelet fluorescence before being passed through a 0.4 mm × 0.1 mm × 28 mm (width × depth × length) micro channel coated with equine type I collagen (Kollagenreagens Horm, Takeda, Austria) at a rate of 80dynes/cm^2^. As blood passed over the channel under shear flow, platelet thrombi were formed, and total surface coverage by thrombus was the variable measured. The channel was mounted onto the stage of an inverted epifluorescence microscope, and a macro on *Image‐Pro Premier* (Media Cybernetics, Rockville, Maryland, USA) was designed for automated calculation of total surface coverage.

**FIGURE 1:**
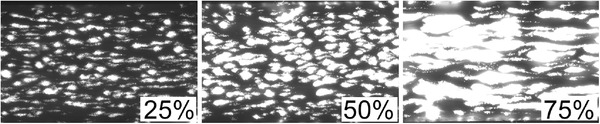
Total surface coverage was the variable measured by the shear flow assay. This percentage value signifies how much of a fixed field of the channel was covered by thrombus, showing as white in this figure. This figure shows the grading with increasing surface coverage, with 25%, 50%, and 75% total surface coverage shown sequentially

Total surface coverage signified thrombus formation, with the median measurement taken over 11 1‐s intervals, 175–185 s, from assay commencement (Figure 1). Total surface coverage by thrombus occurring before the 175–185 s period was deemed to be 100% coverage. A normal range for the surface coverage was developed using 50 normal controls (29 = females, 21 = males, median age 36, range 18–57 years) with normal hemoglobin, platelet count, and hematocrit (Table 1).

**TABLE 1:** Comparing the laboratory values for the 50 normal controls and 22 cTTP patients studied. For sex dependent parameters hemoglobin and hematocrit, no comparative statistics were undertaken as there were too few male cTTP patients (n = 3)

	Normal controls median (range)		cTTP patients median (range)		
	Male	Female	Male	Female	*p* value
**Hemoglobin (g/dL)** ♂ NR: 130–170 ♀ NR: 115–155	150 (132–170)	135 (115–156)	151 (144–155)	129 (104–150)	♂ *♀ = 0.005
HCT (L/L) ♂ NR: 0.37–0.50 ♀ NR: 0.33–0.45	0.46 (0.42–0.50)	0.42 (0.35–0.48)	0.43 (0.41–0.45)	0.38 (0.32–0.43)	♂ *♀ <0.0001
**Platelets (x10^9^/L)** ♂/♀ NR: 150–400	247 (160–378)		266 (133–371)		0.65
**VWF activity (%)** ♂/♀ NR 50–187	102 (48–184)		184 (83–335)		<0.0001
**VWF antigen (%)** ♂/♀ NR 50–160	103 (40–191)		123 (54–276)		0.0009
**ADAMTS13 (IU/dL)** ♂/♀ NR 60–146	113 (68–117)		<5 (<5–8)		<0.0001

### 2.4 VWF analysis

**FIGURE 2:**
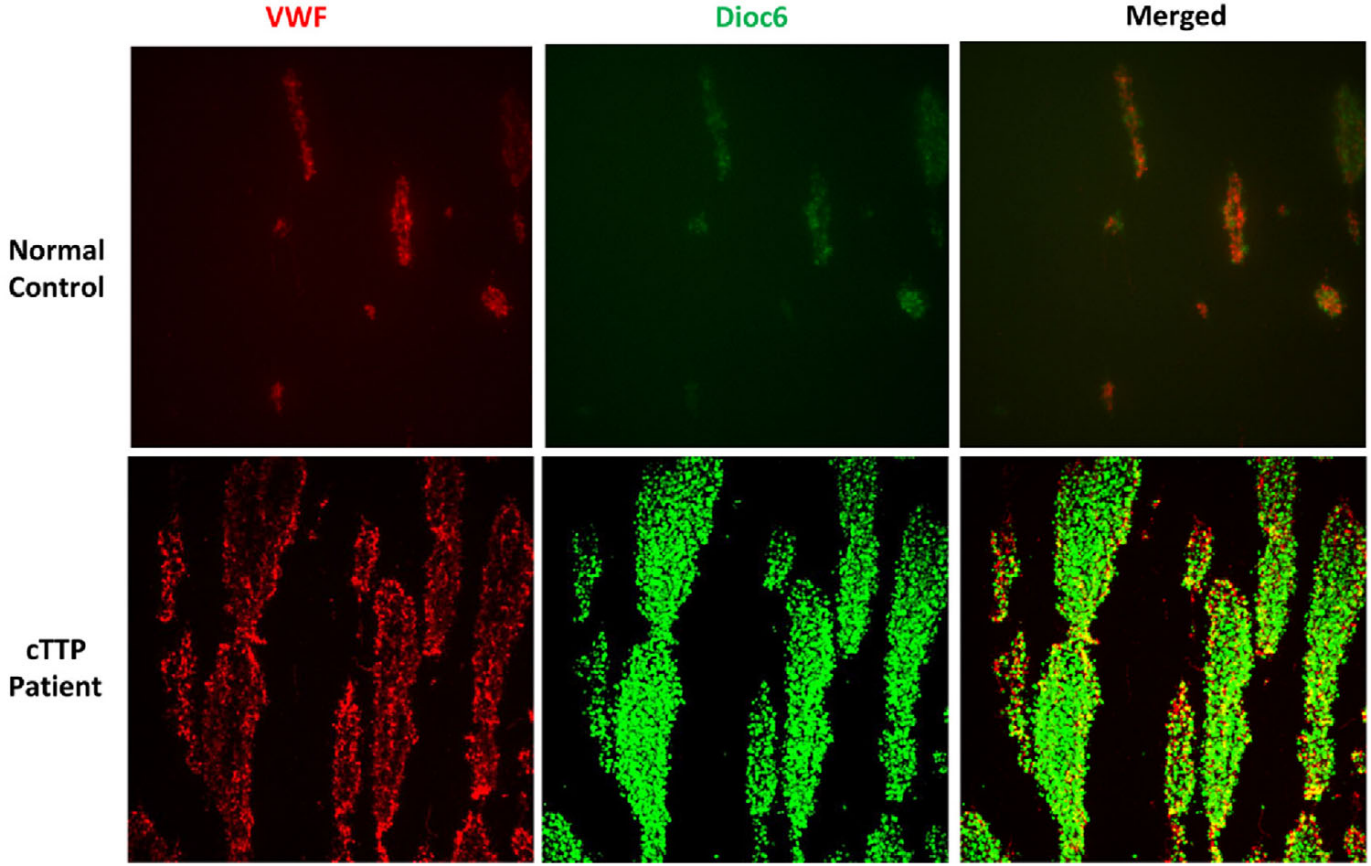
Total surface coverage was the variable measured by the shear flow assay. This percentage value signifies how much of a fixed field of the channel was covered by thrombus, showing as white in this figure. This figure shows the grading with increasing surface coverage, with 25%, 50%, and 75% total surface coverage shown sequentially

Composite pictures of formed thrombi highlighting VWF and platelets were made and used to compare the difference in thrombi in cTTP patients before and after treatment (Figure 2). Samples were fixed within the microchannels in formaldehyde after the shear flow assay was completed. Fixed samples were additionally stained for VWF using a previously described method [22] utilizing polyclonal rabbit anti‐human VWF as the primary antibody and enzyme labeled donkey anti‐rabbit IgG as the secondary antibody to create a visible color reaction. Samples were analyzed under a spinning disc confocal microscope, modified by the addition of an electron multiplying charged couple device camera to increase single photon detection sensitivity. A further automated macro on *Image‐Pro Premier* was designed to quantify the proportions of VWF seen and length of VWF strings.

VWF multimer analysis was also undertaken using simultaneous fresh blood samples, for high and low‐resolution vertical SDS‐Agarose gel electrophoresis to correlate findings from the flow‐based method. This analysis was undertaken in Hamburg, Germany, using a previously published method [23]. In cTTP patients the ultra large multimers are of special interest, and so the large multimers (oligomers > 10) were subdivided into large (oligomers 11–15) and ultra large multimers (oligomers > 15).

VWF antigen (normal range (NR): 50–160%) and activity (NR: 50–187%) were performed via a *Sysmex CS2100i* analyzer (Sysmex Corporation, Kobe, Japan) using a *Siemens INNOVANCE*® VWF kit (Siemens, Marburg, Germany) [24]. An immunoturbidimetric method utilizing a recombinant gain‐of‐function GP1b fragment was the basis of the activity assay.

### 2.5 Patient therapy

Prophylactic treatment was with either intermediate purity factor VIII concentrate (BioProducts Laboratory, Elstree, Herts, UK (BPL)‐8Y) at approximately 15–20 IU/kg given fortnightly, or PI at a volume of 10 mL/kg given every 1–2 weeks depending on laboratory parameters and patient symptoms [4]. Citrate whole blood samples were taken 30 min before and after treatment. Repeat measurements were undertaken after patients initiated aspirin 75 mg once daily for at least 10 days in conjunction with standard PI. If patients could not tolerate aspirin, clopidogrel 75 mg once daily was commenced as an alternative.

To confirm the correlation between surface coverage and ADAMTS13 levels, further experiments were undertaken, adding recombinant ADAMTS13 (rADAMTS13) in vitro to all pre‐treatment citrated blood samples, and three different ADAMTS13 activity levels were assessed: 47 IU/dL, 93 IU/dL, and 140 IU/dL (NR: 60–146 IU/dL). Samples were incubated for 15 min after the addition of rADAMTS13.

All patients were included in the statistical analyses, performed using *Graphpad Prism 6* (GraphPad Software Inc., La Jolla, California, USA) and *SPSS Statistics for Macintosh*, version 22 (IBM Corp, Armonk, New York, USA).

## 3 RESULTS

Fifty normal controls (29 female, 21 male, median age 36, range 18–57 years) were analyzed. Twenty‐two patients with cTTP were included (19 females, 3 male) with a median age of 33 years (16–69 years) at the time of analysis. The median baseline platelet count was 266 × 10^9^/L (133 – 371 × 10^9^/L). Blood count parameters are listed in Table 1.

### 3.1 Platelet surface coverage

All controls had hemoglobin, hematocrit, platelet count, VWF activity, VWF antigen, and ADAMTS13 activity levels within the normal range. The surface coverage normal range was 6–39%. The intra‐ and inter‐assay coefficients of variation were 12% and 13%, respectively. Surface coverage by thrombus formation was significantly higher in patients with cTTP than in normal controls (*p* < 0.0001) with a median pre‐treatment surface coverage of 89% (range: 47–100%) compared to 23% in normal controls (range: 6–39%). Using one‐way MANOVA multivariate analysis, ADAMTS13 activity levels (*p* < 0.0001), VWF activity levels (*p* < 0.0001), and VWF antigen levels (*p* = 0.003) were found to significantly influence the degree of surface coverage.

Using a custom macro to calculate surface coverage, thrombi formed in cTTP patients were larger than those formed in normal controls with both increased VWF adhesion to collagen and increased VWF size noted in the cTTP cases (Figure 2). In cTTP patients, six times more VWF was seen per field than that identified in normal controls (Figure 2).

### 3.2 Treatment

Twenty patients received prophylaxis, and two patients received on demand treatment. Of those who received prophylaxis, two had independent periods of treatment with PI and BPL‐8Y. Therefore, a total of seventeen patients were treated with PI and five patients with BPL‐8Y. The two patients who switched from PI to BPL‐8Y did so after experiencing reactions to plasma. Of these, one was able to resume PI with no symptom reoccurrence.

**FIGURE 3:**
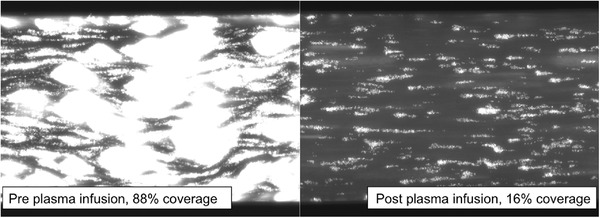
A demonstration of the thrombus formation seen in the shear flow assay. Whole blood is passed over a collagen‐coated chip under shear flow. Increased thrombus formation is seen in cTTP patients when compared to normal controls. Quantification of surface coverage by thrombus formation is calculated at a fixed time point and used as a measure of severity and/or treatment response. This figure demonstrates the same patient before plasma infusion and afterwards. This patient was receiving oral aspirin in addition to prophylactic plasma infusion

PI decreased surface coverage from a median pre‐treatment coverage of 89–44% (*p* = 0.0005) post‐treatment (Figure 3). After administration of BPL‐8Y, surface coverage improved from a median of 65–41%, although this was not statistically significant (*p* = 0.095). In 40% of patients, surface coverage returned to the normal range with BPL‐8Y treatment compared to 50% with PI (*p* > 0.99). However, these improvements did not persist until the next prophylactic therapy for any patient. One hundred percent of patients whose surface coverage normalized with prophylaxis saw it return pre‐treatment levels by the time of their next infusion, regardless of whether they had prophylaxis weekly or fortnightly, with PI or BPL‐8Y.

Nineteen patients subsequently started on aspirin 75 mg daily. Three patients who experienced gastrointestinal symptoms switched to clopidogrel 75 mg daily. The addition of an antiplatelet agent to regular PI prophylaxis reduced pre‐treatment surface coverage further (median 50% [range: 18–66%] compared to 89% without antiplatelet therapy [range: 47–100%], [*p* < 0.0001]). This reduction persisted after treatment with post‐treatment surface coverage of 19% (range: 11–36%) after both antiplatelet PI compared to 44% (range: 25–66%) for PI alone (*p* = 0.0011). One hundred percent of patients receiving an antiplatelet medication in conjunction with PI saw surface coverage return to the normal range, compared to 50% of patients with PI alone (*p* = 0.04). Antiplatelet therapy, combined with regular PI prophylaxis, resolved non‐overt symptoms such as headache, lethargy, and abdominal pain in 100% of patients compared to 74% with PI alone (*p* = 0.046).

A dose dependent response was seen on in vitro addition of recombinant ADAMTS13 to citrated whole blood pre‐prophylaxis samples. The median levels of surface coverage were 53% (range: 18–100%), 46% (range: 14–100%), and 33% (range: 17–75%) for rADAMTS13 levels of 47 IU/dL, 93 IU/dL, and 140 IU/dL, respectively.

### 3.3 VWF activity, antigen, and multimers

VWF activity levels were higher in cTTP patients with a median VWF activity of 184% compared to 102% for normal controls (*p* < 0.0001). VWF antigen levels were also significantly higher (cTTP median 123%, normal controls median 103%, *p* = 0.0006). VWF activity levels in cTTP patients did not change with PI (post infusion median 166%, *p* = 0.69), BPL‐8Y (post‐8Y median 195%, *p* = 0.97) or antiplatelet therapy (post‐antiplatelet median 185%, *p* = 0.70). Comparative results excluding aspirin administration are summarized in Table 2.

**TABLE 2:** ADAMTS13, VWF activity, and VWF antigen levels were compared at baseline and after administration of factor 8Y concentrate or fresh frozen plasma

Treatment status	ADAMTS13 (IU/dL)	VWF activity (%)	VWF antigen (%)	Surface coverage (%)
Pre‐treatment (*n* = 20)	<5 (<5–12)	184.0% (101.8–290.4)	123.2 (72.5–239.4)	89% (47–100%)
Post‐8Y (*n* = 5)	<5 (<5–11)	194.7% (117.2–270.5)	154.8 (141.2–219.0)	41% (31–62%)
Post‐FFP (*n* = 17)	24 (17–28)	166.3% (111.8–235.4)	125.6 (95.3–178.1)	44% (25–66%)

**FIGURE 4:**
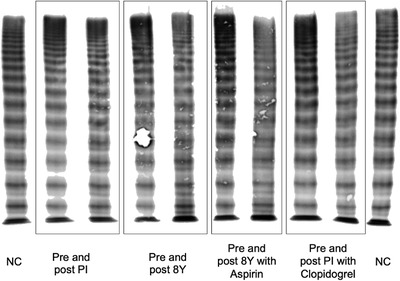
VWF multimer patterns from normal controls (1,2), and a single cTTP patient who was treated with plasma infusion, BPL‐8Y, aspirin, and clopidogrel. Results are explained in Table 3

Serial VWF multimer analyses were undertaken in three cTTP patients. These showed the classic banding pattern seen in patients with cTTP, with an increase in large and ultra large multimers [25]. One patient over time received PI, BPL‐8Y, aspirin, and clopidogrel; and the effect of each treatment was reviewed by multimer analysis (Table 3, Figure 4). A decrease in large and ultra‐large multimers was seen after all ADAMTS13 containing therapies in the low resolution gel with additional enhancement of proteolytic sub‐bands.

**TABLE 3:** To confirm the results seen by the shear flow assay, citrated whole blood samples were also analyzed for VWF multimer levels by SDS‐agarose gel electrophoresis. This confirmed that plasma infusion and BPL‐8Y reduced the proportion of ultra‐large and large multimers seen. Caution is required by interpreting the BPL‐8Y post‐treatment results as these include quantification of the donor FVIII contained in this

	Ultra‐large (%)	Large (%)	Intermediate (%)	Small (%)
**Normal control 1**	14.1	22.3	35.4	28.3
**Normal control 2**	13.5	20.6	36.5	29.3
**Pre PI**	25.7	20.9	29.7	23.7
**Post PI**	17.2	17.6	35.7	29.5
**Pre BPL‐8Y**	25.1	24.6	29.3	21.0
**Post BPL‐8Y**	13.9	18.5	30.0	37.5
**Pre BPL‐8Y + Aspirin**	23.4	23.2	32.2	21.1
**Post BPL‐8Y + aspirin**	10.2	18.5	34.8	36.5
**Pre PI + clopidogrel**	20.8	24.9	33.0	21.3
**Post PI + clopidogrel**	10.5	21.3	38.4	29.8

## 4 DISCUSSION

Management of patients with cTTP has been hindered by the lack of an evidence base, but emerging data suggest that patients have non‐overt symptoms and adverse events related to end organ damage if untreated [4]. There is, therefore, a need for methods to understand the impact of disease and assess adequate treatment response.

This study utilizes a dynamic assay to model VWF‐mediated platelet adhesion and aggregation and demonstrates VWF‐platelet complexes, which could potentially reflect the in vivo impact in cTTP patients who describe non‐overt symptoms. This is the first study to demonstrate ongoing evidence of microvascular thrombosis despite normal routine blood counts and the effect of ADAMTS13 replacement therapies. We demonstrate that despite normal blood counts, patients with cTTP have significantly increased VWF adsorption to collagen and subsequent increased platelet adhesion to VWF multimers causing in vitro thrombus formation. Additionally, we show that with ADAMTS13 replacement therapies, thrombus formation can be reduced. The presence of larger VWF multimers was confirmed by SDS‐Agarose gel electrophoresis multimer analysis and was in keeping with the recognized multimeric pattern seen in cTTP [26]. FFP infusion and BPL‐8Y both lead to reduction in the quantity of ultra large and large circulating VWF multimers, the latter likely due to the product's exogenous factor VIII binding to the patient VWF and enhancing its susceptibility to proteolysis by the small amount of infused ADAMTS13. PI is the most commonly used prophylactic treatment, and while all patients saw a decrease in surface coverage, only 50% of patients saw it decrease to a level comparable to normal controls. Similar results were seen with an intermediate purity factor VIII concentrate, although the latter therapy did not achieve statistical significance for efficacy, in part because of the small sample size for patients receiving it. Additionally, the pre‐treatment surface coverage levels seen in patients who were treated with BPL‐8Y were lower than those treated with PI, differences which suggest caution is needed when comparing the results of these two therapies.

While it is encouraging to note that in vivo prophylaxis is seen to reduce in vitro thrombus formation, improvements were short‐lived, even with weekly treatment, and in all patients, surface coverage returned to their high baseline levels by the time of their next therapy, suggesting the current treatment options may not be adequate. There is a limited increment of ADAMTS13 achieved with PI [11], and there remains indecision around what ADAMTS13 activity level should be achieved. It is possible that in the future, recombinant ADAMTS13 (rADAMTS13) given at an adequate dosing interval will improve upon the current options and show a further sustained reduction in surface coverage compared to PI and BPL‐8Y. From the Phase 1 rADAMTS13 in cTTP study [27], a 40 U/kg dose gave a median peak plasma concentration (C_max_) of 94 IU/dL, which cannot be achieved with PI or current factor concentrates.

We hypothesize that cTTP patients may require higher ADAMTS13 levels than currently achieved because of the presence of circulating ultra large and large VWF multimers and the increased propensity of VWF to bind to collagen, causing exaggerated adhesion and aggregation of platelets and resulting more extensive thrombus formation demonstrated in this work. It is likely that this explains the non‐overt symptoms seen in cTTP and may well be relevant to the high vascular complication rates recently described [4, 6]. Going forward, in larger cohorts, it would be interesting to replicate these data in patients receiving rADAMTS13 therapy and perhaps allow for dose adjustments once preexisting UL‐VWF multimers have been cleaved.

An important finding in this study is the impact of antiplatelet therapy. This further reduced thrombus formation in patients with cTTP and synergistically improved results alongside all the ADAMTS13 replacement therapies. This was coupled with a further clinical improvement in the non‐overt symptoms reported by patients such as headache, lethargy, and abdominal pain. Antiplatelet therapy is not typically offered to cTTP patients. A caveat to this finding is that the antiplatelet will, in part, have inhibited platelets which were activated by collagen, and collagen exposure secondary to endothelial injury is not a major factor of TTP pathogenesis. Platelet activation is however seen secondary to a number of other processes such as VWF activation and thrombin exposure. While the effect would be best assessed by comparing the effect of antiplatelets in normal controls and cTTP patients, we believe that in patients at increased risk of microthrombi formation, there is validity in the findings despite the aforementioned limitation.

The results seen in this assay were corroborated by SDS‐Agarose gel electrophoresis multimer analysis, which found increased large and ultra‐large multimers in cTTP patients which then reduced with treatment, in a similar manner to the improvement in surface coverage by thrombus seen with the shear flow assay. In addition to comparable results with an established assay, the shear flow method has the benefit of giving more immediate results. There is also ample scope to automate this process fully, making it easier to interpret results without the expert analysis required for multimer studies.

There are limitations to this study. While the sample size is large for such a rare disorder, it is not sufficient to draw definitive conclusions on the effect of different treatments. However, the results show a notable increase in thrombus formation in cTTP with a clear improvement with therapy. As such, there is an argument to prophylactically treat all patients once a cTTP diagnosis is made, based on the assumption that there is the potential for ongoing microthrombi without therapy. Statistical significance was not achieved when evaluating the level of improvement seen with patients treated with BPL‐8Y, in part because of lower pre‐treatment levels of thrombus formation when compared to the PI cohort. In this study, patients preexisting prophylactic management plan was not changed, and so we hypothesize that the lower baseline levels of surface coverage in BPL‐8Y patients can be explained by a possible study bias with less symptomatic adult patients more likely to be treated with BPL‐8Y ahead of PI. Finally, these results depend on the use of collagen‐coated channels. While collagen is an intrinsic part of hemostasis [28, 29] and used as a standard in hemostatic [30] and microfluidic [31] assays, physiologically, it is part of the sub‐endothelial matrix and only exposed to blood flow during injury. The degree of thrombus formation is therefore increased compared to normal, in vivo laminar blood flow over endothelial cells. Small injuries to vessels are a common occurrence in normal vasculature; therefore the use of collagen mimics the physiological milieu to an exaggerated extent rather than directly replicating what is happening in vivo.

In conclusion, we utilize a dynamic assay to demonstrate that increased in vitro platelet‐VWF adhesion occurs despite normal blood counts in cTTP patients outside of acute episodes suggesting an increased potential to form microvascular thrombi. This expands our understanding of the impact of severe ADAMTS13 deficiency associated with non‐overt microvascular thrombosis, escalating our understanding of disease pathophysiology. Prophylactic therapy with ADAMTS13 rich products helps to ameliorate thrombi, but the dose and frequency needs optimizing, for which this assay, in conjunction with ADAMTS 13 assays, could tailor therapy. Consideration should also be given to starting patients on low dose antiplatelet agents, which were well tolerated and led to symptomatic improvement when combined with ADAMTS13 replacement therapy.

## AUTHOR CONTRIBUTIONS

Ferras Alwan and Chiara Vendramin developed the assay, collected data, wrote the paper, and undertook laboratory testing. Ulrich Budde undertook laboratory testing and reviewed the manuscript. Ri Liesner and Alice Taylor collected data. Mari Thomas collected data and reviewed the manuscript. Bernhard Lämmle reviewed the manuscript, and Marie Scully was senior author, collected data, and reviewed the manuscript.

## CONFLICT OF INTEREST

Ri Liesner: Bayer: consultancy, research funding; Sobi: speakers bureau; Roche: research funding; Baxalta: consultancy, research funding; Novo Nordisk: research funding, speakers bureau; Octapharma: consultancy; other: clinical study investigator for NuProtect study (Octapharma sponsored), research funding, speakers bureau. Mari Thomas: member of advisory boards for Sanofi and Bayer. Bernhard Lämmle: lecture fees from Alexion, Bayer, Roche, Sanofi and Siemens; member of advisory board for Ablynx and a data safety monitoring board for Takeda. Marie Scully: member of advisory boards for and speaker fees from Alexion, Novartis, Sanofi and Takeda. PhD project funding from Shire.
